# Supplementary material for: Glacial heterogeneity in Southern Ocean carbon storage abated by fast South Indian deglacial carbon release
Source: Nat Commun. 2020 Dec 3;11:6192. doi: 10.1038/s41467-020-20034-1 (PMC7712879; doi:10.1038/s41467-020-20034-1)
Supplement: Supplementary file 1 — Supplementary Information [file 41467_2020_20034_MOESM1_ESM.pdf]

Supplementary Information

Glacial heterogeneity in Southern Ocean carbon storage abated by fast South Indian deglacial carbon release by Gottschalk et al.

Contains: Supplementary Figure 1-11

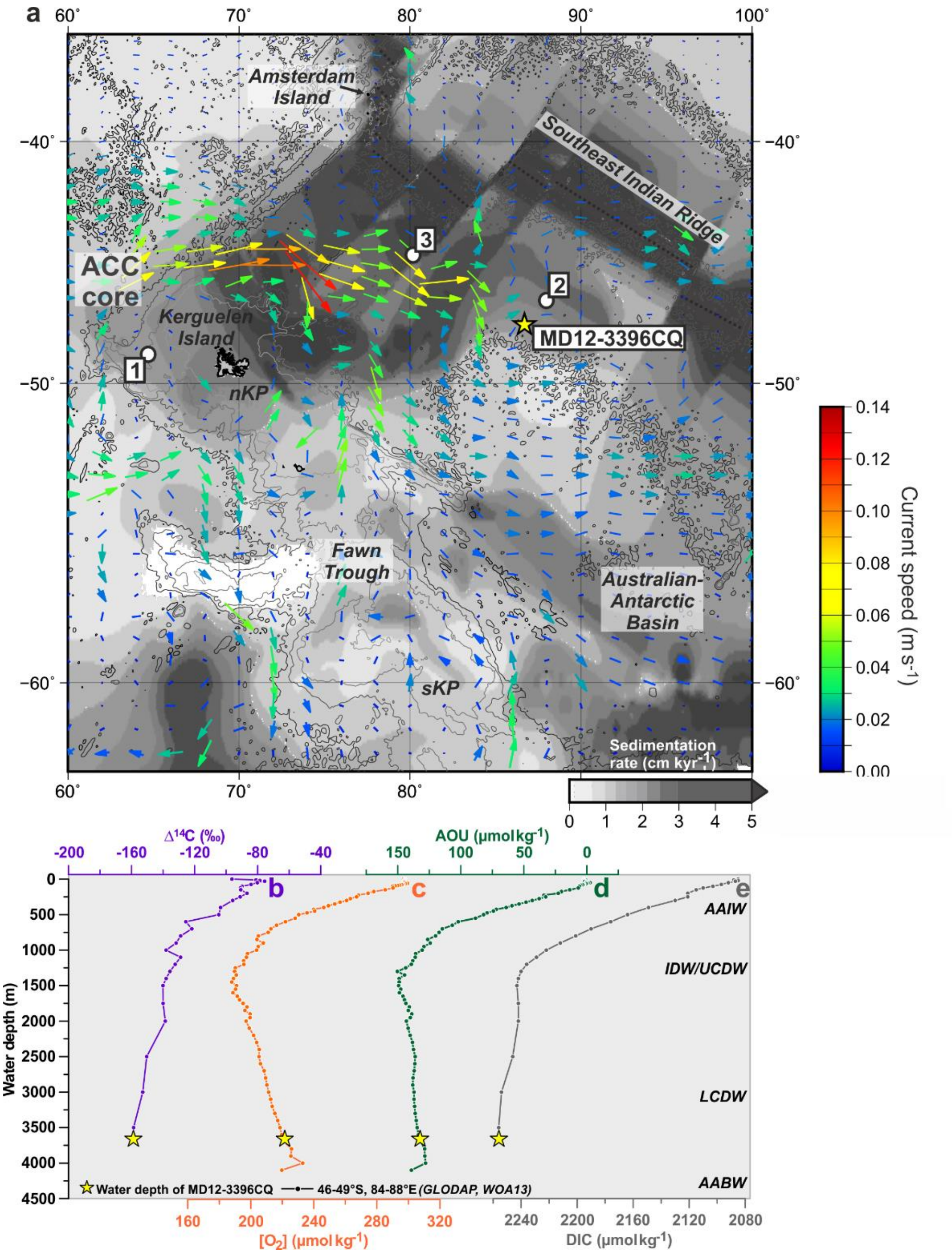

Supplementary Figure 1. South Indian study region near Kerguelen Plateau. (a) Contoured bathymetric map showing the average speed (colors and vector lengths) and direction (vectors) of ocean currents at mid-depth (1.0-3.5 km) based on the Global Ocean Data Assimilation System (GODAS) database (https://psl.noaa.gov/data/gridded/data.godas.html), i.e., the pathway of the Antarctic Circumpolar Current (ACC) core at mid-depth[1]. The study area is located within a prominent sediment drift south of the Southeast Indian Ridge, as indicated by simulated sedimentation rate patterns (grey)[2]. The drift deposit is formed by sediment release from the ACC just downstream of the location of maximum current speeds north of the northern and southern Kerguelen Plateau (nKP and sKP, respectively) owing to a drop in ACC flow speed[2–4]. (b) Bomb-corrected ocean ^{14}C levels[5] (in $\Delta^{14}C$ notation, i.e. relative to the 1950 atmosphere), (c) seawater oxygen concentrations ($[O_2]$)[6], (d) apparent oxygen utilization (AOU)[6], and (e) total dissolved inorganic carbon (DIC) levels at the core site[5]. Note that x-axes in (d) and (e) are inverted. The location of the study core MD12-3396CQ is indicated by a star. Other cores highlighted in the main text are marked with a circle (1: MD12-3394, 2: MD02-2488 and 3: MD12-3401CQ). Right-hand labels in the lower panel indicate major water masses in our South Indian study area[7–12]: AAIW – Antarctic Intermediate Water, IDW – Indian Deep Water, UCDW – Upper Circumpolar Deep Water, LCDW – Lower Circumpolar Deep Water, AABW – Antarctic Bottom Water.

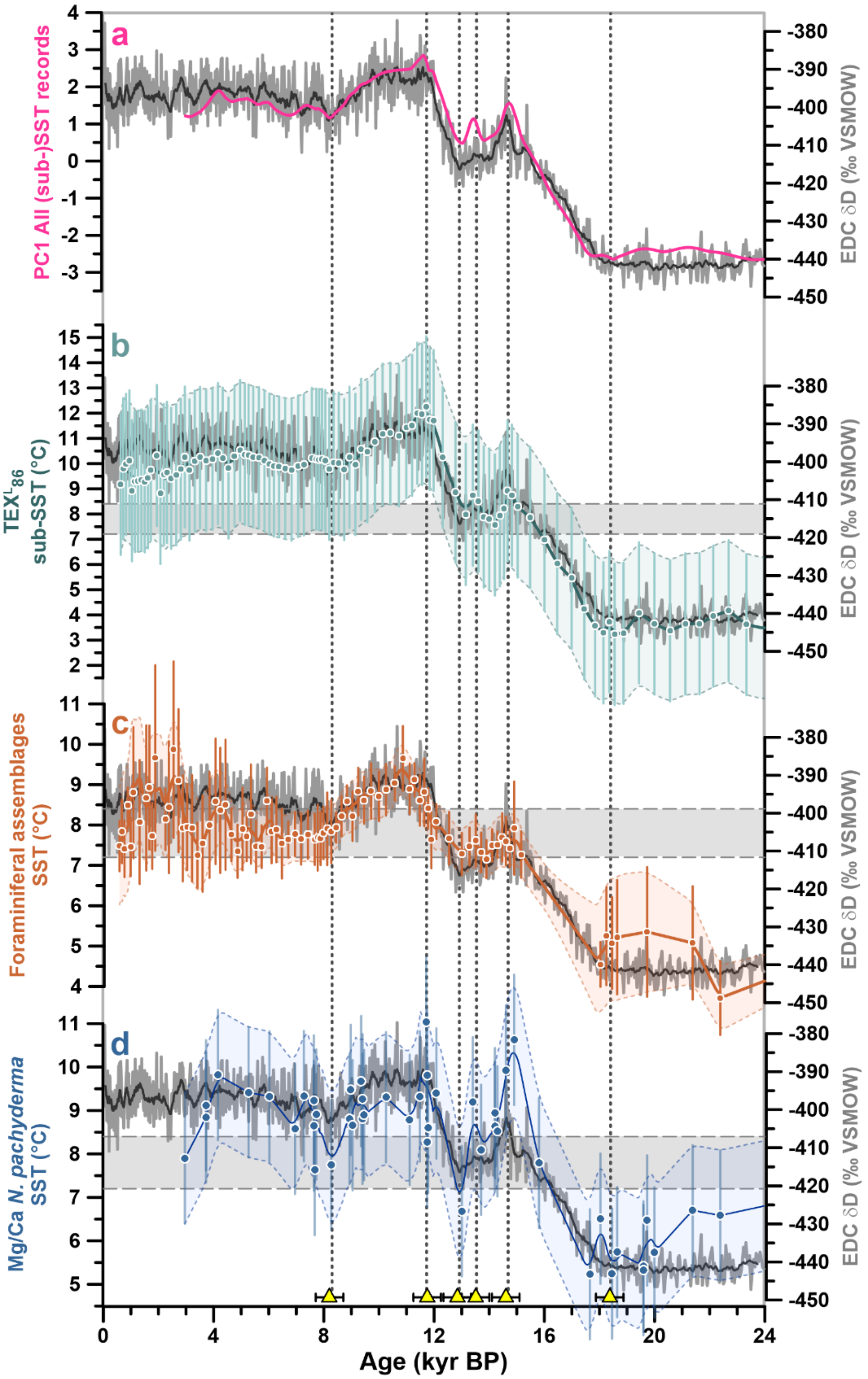

Supplementary Figure 2. (Sub-)sea surface temperature variability recorded in MD12-3396CQ during the last deglaciation. (a) First principal component (PC1) of our (sub-)sea surface temperature (SST) records, (b) TEX$^{L}_{86}$-based sub-SST estimates based on the sub-surface calibration of ref. 13 (1σ-standard deviation (SD) is assumed to be 2.8°C), (c) summer SST derived from planktonic foraminiferal assemblage changes using a recent Southern Ocean calibration[14] (mean 1σ (SD)-uncertainty is 1.1°C), and (d) temperature-converted[15] Mg/Ca ratios of the planktonic foraminiferal species *Neogloboquadrina pachyderma* (1σ (SD)-uncertainty is assumed to be 1.5°C). Solid lines indicate 1 kyr-running averages. Envelopes indicate the 1σ (SD)-uncertainty range (smoothed). Grey horizontal bars indicate the modern annual SST variability at the study site (0-50 m average, World Ocean Atlas 2013)[16]. All (sub-)SST datasets are superimposed on Antarctic temperature variability, represented by δD variations in the EPICA Dome C (EDC) ice core[17] (grey lines). Triangles at the bottom (with vertical lines) indicate tiepoints that were chosen to align (sub-)SST variations at our study site with Antarctic temperature (EDC δD) variability.

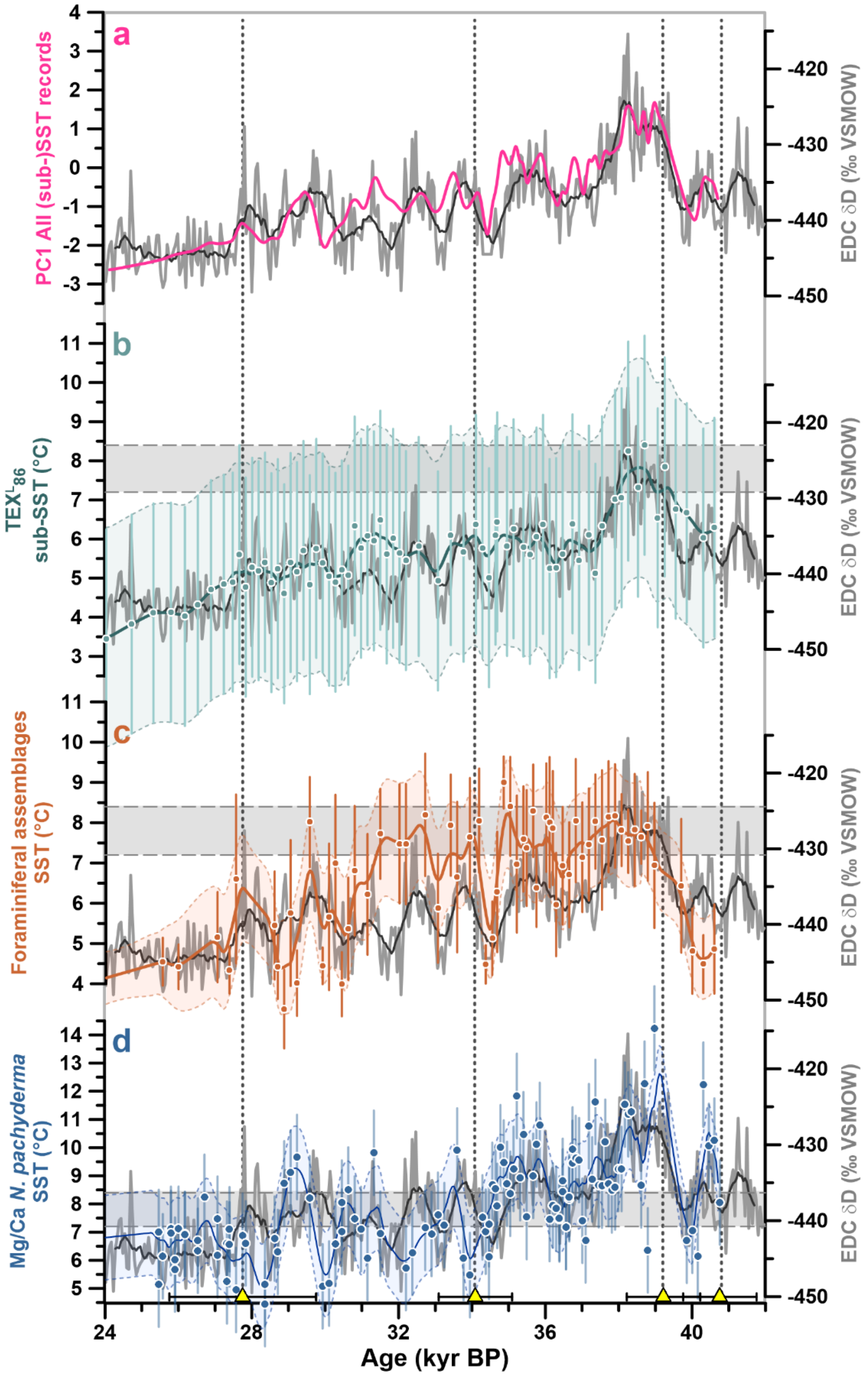

Supplementary Figure 3. (Sub-)sea surface temperature variability recorded in MD12-3396CQ during late Marine Isotope Stage 3. (a) First principal component (PC1) of our (sub-)sea surface temperature (SST) records, (b) TEX^{L}_{86}-based sub-SST estimates based on the sub-surface calibration of ref. 13 (1σ-standard deviations (SD) is assumed to be 2.8°C), (c) summer SST derived from planktonic foraminiferal assemblage changes using a recent calibration[14] (mean 1σ (SD)-uncertainty is 1.1°C), and (d) temperature-converted[15] *N. pachyderma* Mg/Ca ratios (1σ (SD)-uncertainty is assumed to be 1.5°C). Solid lines indicate 1 kyr-running averages. Envelopes indicate the 1σ (SD)-uncertainty range (smoothed). Grey horizontal bars indicate the modern annual SST variability at the study site (0-50 m average, World Ocean Atlas 2013)[16]. All (sub-)SST datasets are shown superimposed on the EPICA Dome C (EDC) δD record[17] (grey lines). Triangles at the bottom (with vertical lines) indicate tiepoints that were chosen to align (sub-)SST variations at our study site with Antarctic temperature (EDC δD) variability.

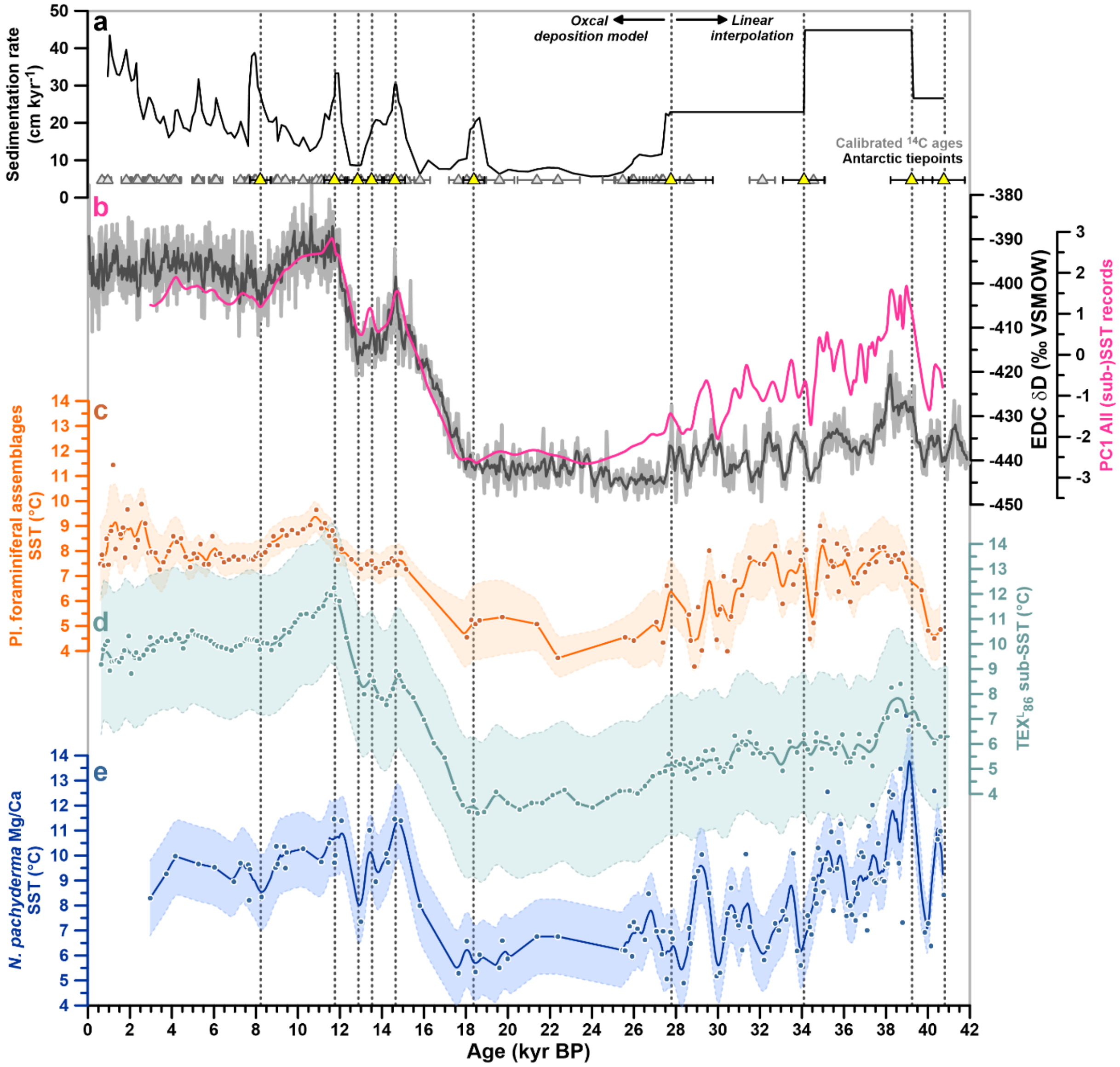

Supplementary Figure 4. Age model and sedimentation rate changes in sediment core MD12-3396CQ. (a) Sedimentation rate changes, (b) first principal component (PC1) of our three (sub-)sea surface temperature (SST) records (pink) and EPICA Dome C (EDC) δD variations[17] (grey), (c) planktic foraminiferal assemblage-based summer SST, (d) TEX^{L}_{86}-based sub-SST estimates, and (e) *N. pachyderma* Mg/Ca-based SST. Solid lines in (c) to (e) indicate 1 kyr-running averages. Envelopes indicate the 1σ (SD)-uncertainty range (smoothed). Yellow triangles (with vertical lines) in (a) indicate tiepoints between (sub-)SST variations and EPICA Dome C (EDC) δD variations (Supplementary Figures 2 and 3). Grey triangles highlight locations of our planktic foraminiferal ^{14}C-dated samples. Uncertainty envelopes shown in c) to e) are identical to those in Supplementary Figures 2 and 3.

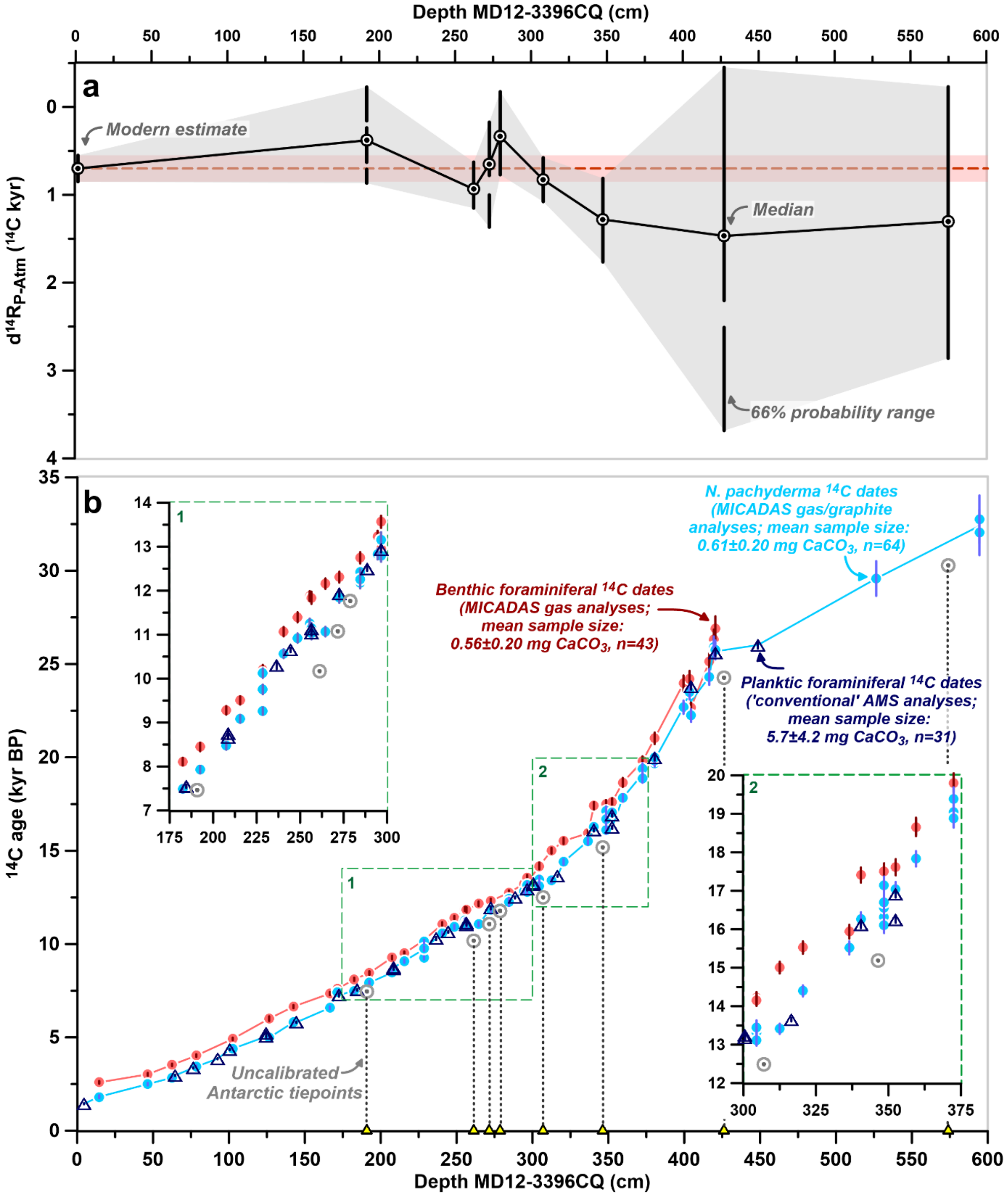

Supplementary Figure 5. Foraminiferal radiocarbon ages versus depth. (a) Surface ocean reservoir ages ($d^{14}R_{P\text{-}Atm}$) at our study site (median, with vertical bars showing the associated 66%-probability range) estimated as the difference between (interpolated) high-resolution planktic foraminiferal ^{14}C dates and our uncalibrated calendar age tiepoints, which were derived from an alignment of (sub-)sea surface temperature (SST) variations with Antarctic temperature recorded by the EPICA Dome C (EDC) δD record (horizontal bar and stippled line show the modern $d^{14}R_{P\text{-}Atm}$ variability in the study region[18–20]), (b) benthic foraminiferal (red) and *N. pachyderma* ^{14}C dates (light blue) obtained with the Bern-Mini Carbon Dating System (MICADAS, gas and graphite ^{14}C analyses), as well as planktic foraminiferal ^{14}C dates obtained with the accelerator mass spectrometer (AMS) system at the University of Paris-Saclay (open triangles; graphite analyses). Error bars in (b) indicate the 1σ-standard deviation. Open grey circles show our Antarctic temperature-based tiepoints that were uncalibrated based on the atmospheric SHCal13 calibration[21] using the radcal.R script[22].

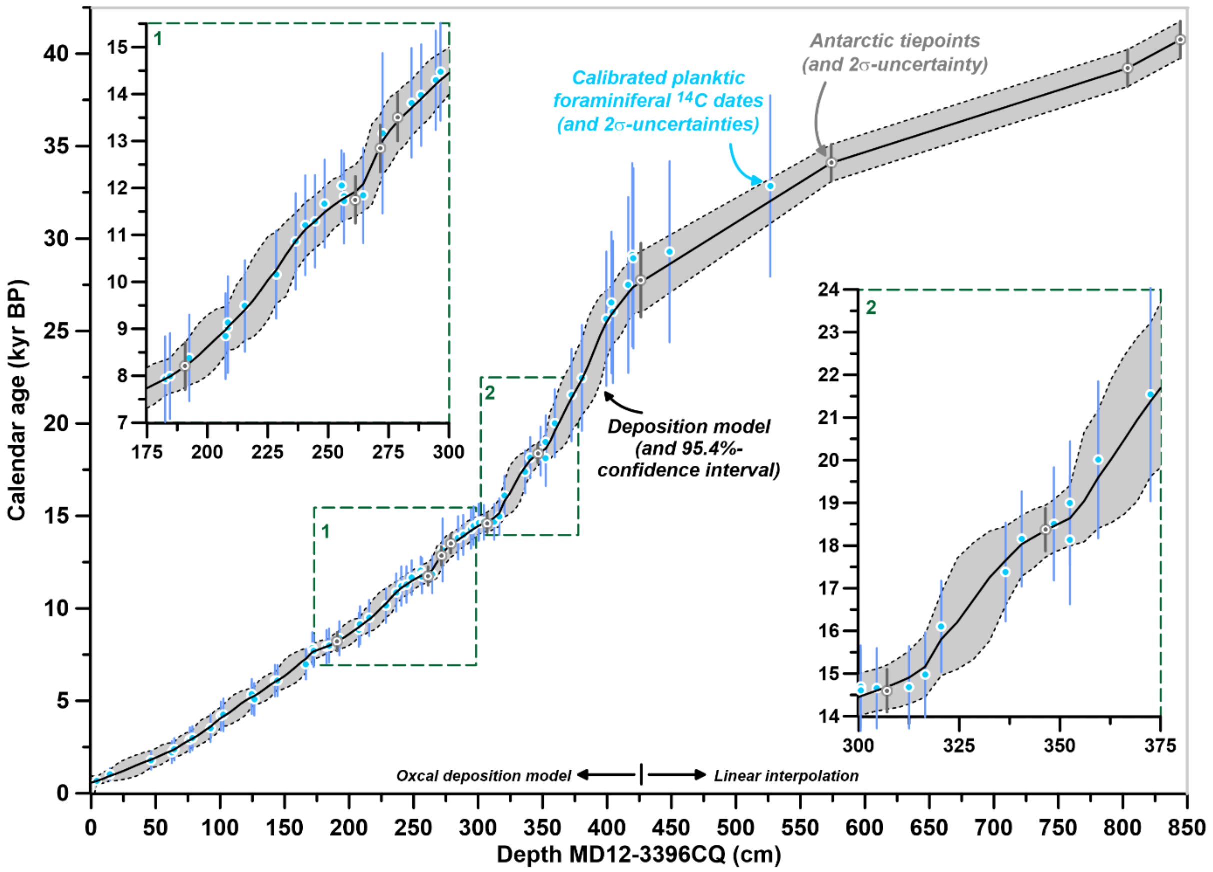

Supplementary Figure 6. Sediment deposition model for core MD12-3396CQ. Our Antarctic temperature-based calendar age tiepoints (grey open symbols) and calibrated planktic foraminiferal ^{14}C dates (light blue) were used as input parameters to calculate a sediment deposition model with *Oxcal* (P_sequence)[23] for MD12-3396CQ (median and 95.4%-probability range are shown as solid line and grey envelope, respectively). The final age model is based on the sediment deposition model of *Oxcal* for the core section with dense dating (<448.5 cm), and linear interpolation for the lower half of the core section with sparse age constraints (>448.5 cm).

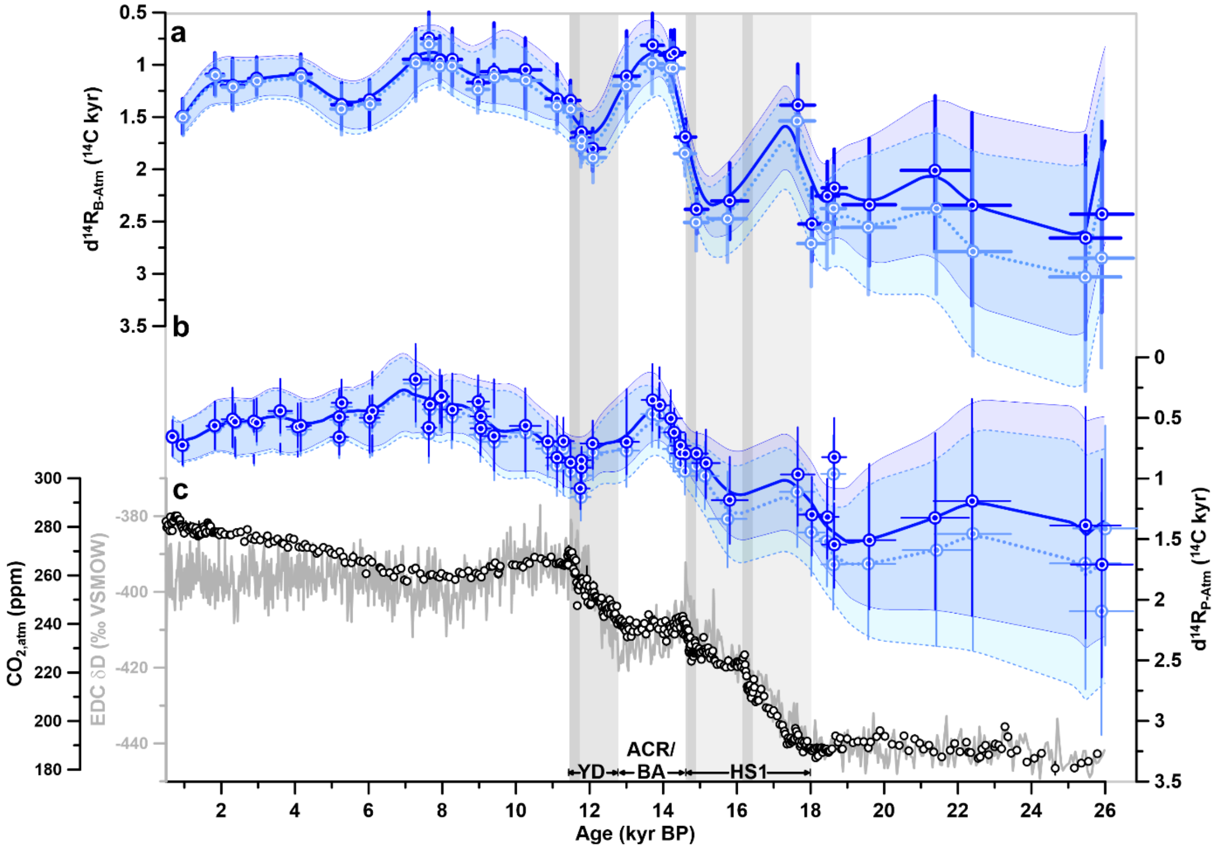

Supplementary Figure 7. Comparison of reservoir age changes in the surface and deep ocean at our study site with and without foraminiferal blank correction. (a) Deep-ocean reservoir age estimates ($d^{14}R_{B\text{-}Atm}$) and (b) surface-ocean reservoir age estimates ($d^{14}R_{P\text{-}Atm}$) based on foraminiferal blank-corrected (light blue, stippled lines) and non-foraminiferal blank-corrected foraminiferal ^{14}C dates (dark blue, solid lines), (c) atmospheric CO_2 ($CO_{2,atm}$) changes (black circles)[24–26] and the EPICA Dome C (EDC) δD record (grey)[17]. Symbols in (a) and (b) indicate the $d^{14}R$ median, horizontal errors bars show the 1σ standard deviation-age uncertainty and vertical error bars highlight the 66%-probability range of our $d^{14}R$ estimates. Envelopes and center lines show 1 kyr-running averages of the minimum and maximum 66%-probability range and the median of our reconstructed $d^{14}R$ values, respectively. Calculations were performed with the radcal.R script[22]. Vertical bars indicate intervals of rising $CO_{2,atm}$ levels. Dark bands highlight periods showing centennial-scale $CO_{2,atm}$ rises[25]. YD – Younger Dryas, ACR – Antarctic Cold Reversal, BA – Bølling-Allerød, HS1 – Heinrich Stadial 1.

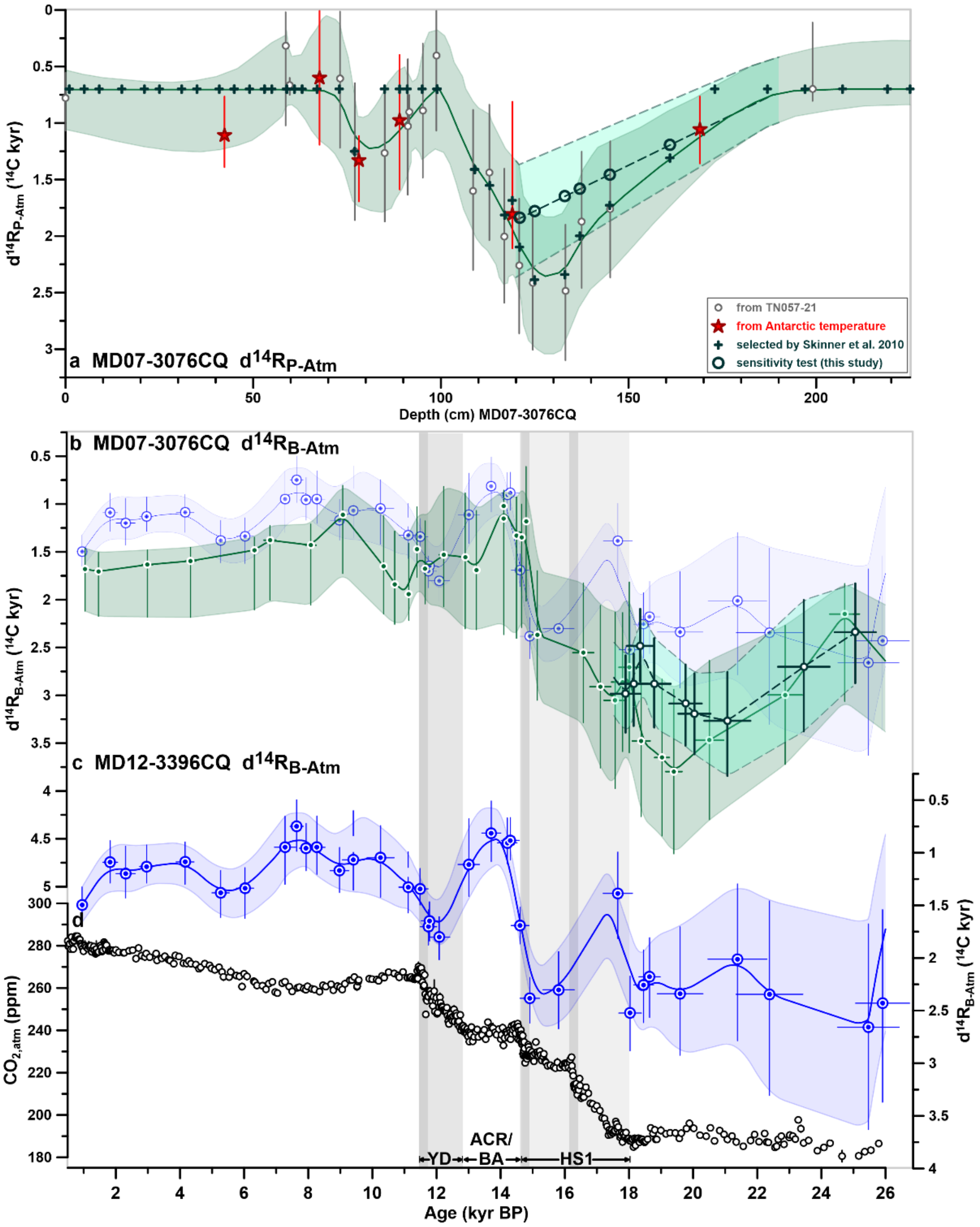

Supplementary Figure 8. Reassessing deep-ocean ventilation age reconstructions in deep South Atlantic sediment core MD07-3076CQ. (a) Surface-ocean reservoir age estimates ($d^{14}R_{P\text{-}Atm}$) values determined in sediment core MD07-3076CQ using a sea surface temperature (SST) alignment to core TN057-21 (grey circles) and to Antarctic temperature (red stars)[27], $d^{14}R_{P\text{-}Atm}$ values applied in ref. 27 are shown as crosses; last glacial $d^{14}R_{P\text{-}Atm}$ values omitting information from TN057-21 are shown as open circles, which are used to test the sensitivity of deep-ocean ventilation ages ($d^{14}R_{B\text{-}Atm}$) variations to $d^{14}R_{P\text{-}Atm}$ changes, in order to compare absolute last glacial deep-ocean ventilation ages between MD07-3076CQ and MD12-3396CQ. (b) $d^{14}R_{B\text{-}Atm}$ estimates in MD07-3076CQ as originally published (green)[27] and with adjusted $d^{14}R_{P\text{-}Atm}$ values (dark green symbols, this study; see open circles and dashed lines in a), (c) $d^{14}R_{B\text{-}Atm}$ estimates in MD12-3396CQ for comparison (replotted in blue in b), and (d) atmospheric CO_2 ($CO_{2,atm}$) variations (black circles)[24–26]. New data in (b) and (c) indicate the reservoir age ($d^{14}R$) median, horizontal error bars show the 1σ standard deviation-age uncertainty and vertical error bars highlight the 66%-probability range of the $d^{14}R$ estimates. Envelopes and center lines show 0.5 kyr (b) and 1 kyr (c)-running averages of the 66%-probability range and the median of reconstructed $d^{14}R$ values, respectively. Calculations were performed with the radcal.R script[22]. Vertical bars indicate intervals of rising $CO_{2,atm}$ levels. Dark bands highlight periods with centennial-scale $CO_{2,atm}$ rises[25]. YD – Younger Dryas, ACR – Antarctic Cold Reversal, BA – Bølling-Allerød, HS1 – Heinrich Stadial 1.

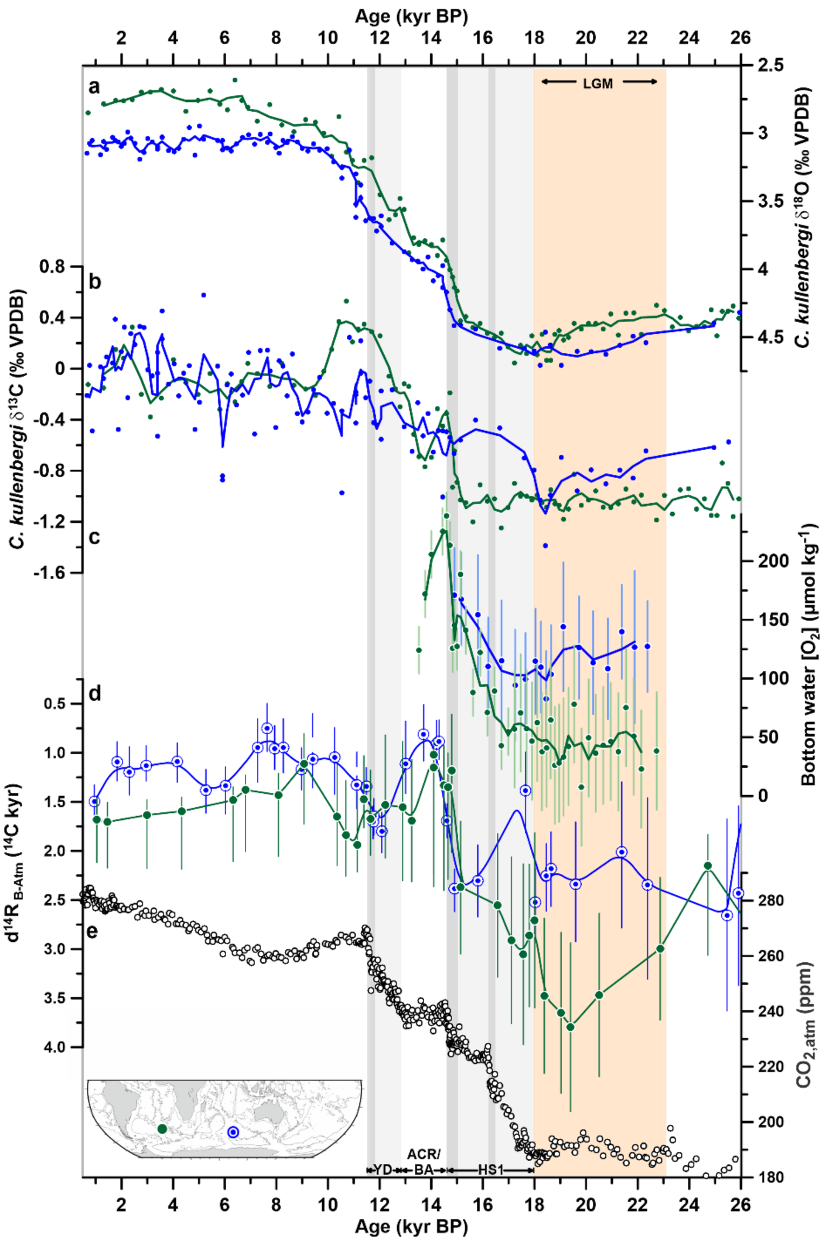

Supplementary Figure 9. Comparison of deep South Atlantic and deep South Indian proxy data. (a) Benthic foraminiferal (*C. kullenbergi*) $\delta^{18}O$ in MD07-3076CQ[28] and MD12-3396CQ (this study), (b) benthic foraminiferal (*C. kullenbergi*) $\delta^{13}C$ in MD07-3076CQ[28] and MD12-3396CQ (this study), (c) bottom water oxygen concentrations ($[O_2]$) reconstructed in MD07-3076CQ[29] and MD12-3396CQ (this study) based on the $\delta^{13}C$ gradient between *G. affinis* and *C. kullenbergi* ($\Delta\delta^{13}C$)[30] with error bars showing the 1 σ-standard deviation, (d) ^{14}C age offsets of benthic foraminifera in MD07-3076CQ[27] and MD12-3396CQ (this study) from the contemporaneous atmosphere ($d^{14}R_{B\text{-}Atm}$) with error bars showing the 66%-probability range, and (e) atmospheric CO_2 ($CO_{2,atm}$) changes[25]. Grey vertical bars indicate intervals of rising $CO_{2,atm}$ levels (darker bands highlight periods of centennial-scale $CO_{2,atm}$ increases[25]). HS1 – Heinrich Stadial 1, ACR – Antarctic Cold Reversal, BA – Bølling Allerød, YD – Younger Dryas. The orange vertical bar highlights the interval of the last glacial maximum (LGM).

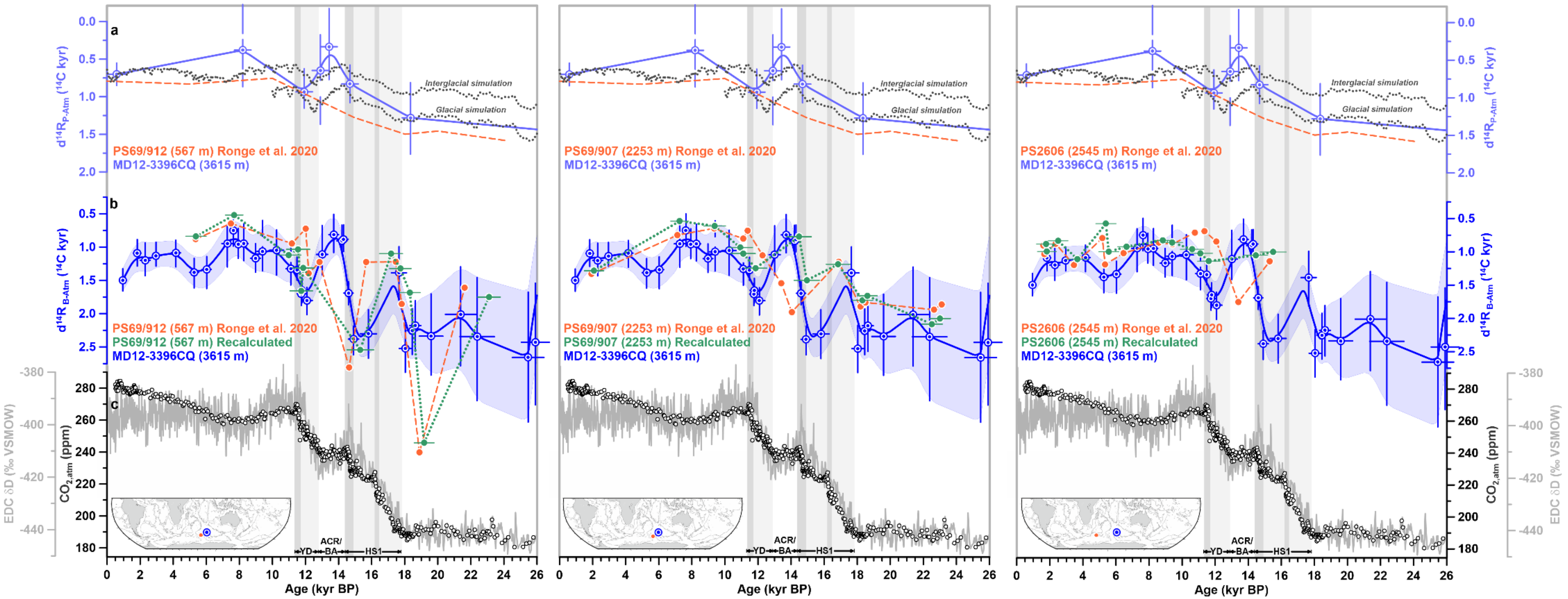

Supplementary Figure 10. Changes in deep-ocean ventilation ages depending on the choice of surface-ocean reservoir ages for published data from the Crozet- and Kerguelen Plateau regions. Sensitivity tests are shown for cores PS69/912 (left), PS69/907 (middle) and PS2606 (right) from ref. 31, and are compared against proxy-data from MD12-3396CQ (this study). For locations of cores, see inset maps. (a) Surface-ocean reservoir age ($d^{14}R_{P\text{-}Atm}$) constraints from MD12-3396CQ (this study, light blue, error bars as in Fig. 7b) based on a stratigraphic alignment between (sub-)sea surface temperature and Antarctic temperature variations. $d^{14}R_{P\text{-}Atm}$ values for the Kerguelen Plateau region inferred by ref. 31 are shown in orange. Simulated $d^{14}R_{P\text{-}Atm}$ changes between 80-100°E and 45-50°S at 25 m water depth as obtained with an ocean general circulation model forced by variations in atmospheric ^{14}C levels ($\Delta^{14}C$) under both interglacial (PD simulation) and glacial boundary conditions (GS simulation) are shown as stippled lines for comparison[32]; (b) ^{14}C age offsets of benthic foraminifera in MD12-3396CQ from the contemporaneous atmosphere, $d^{14}R_{B\text{-}Atm}$ (blue, error bars as in Fig. 7a); $d^{14}R_{B\text{-}Atm}$ reconstructed for PS69/912, PS69/907 and PS2606 as published in ref. 31 (orange) using $d^{14}R_{P\text{-}Atm}$ values shown in orange in (a) and recalculated with $d^{14}R_{P\text{-}Atm}$ constraints from MD12-3396CQ (green, horizontal error bars show 1σ-standard deviations) shown in light blue in (a), calculations follow procedures outlined in ref. 31; (c) atmospheric CO_2 ($CO_{2,atm}$) variations (circles)[25] and EPICA Dome C (EDC) δD changes (grey line)[17,33]. Grey vertical bars indicate intervals of rising $CO_{2,atm}$ levels (darker bands highlight periods with centennial-scale $CO_{2,atm}$ increases[25]). HS1 – Heinrich Stadial 1, ACR – Antarctic Cold Reversal, BA – Bølling Allerød, YD – Younger Dryas.

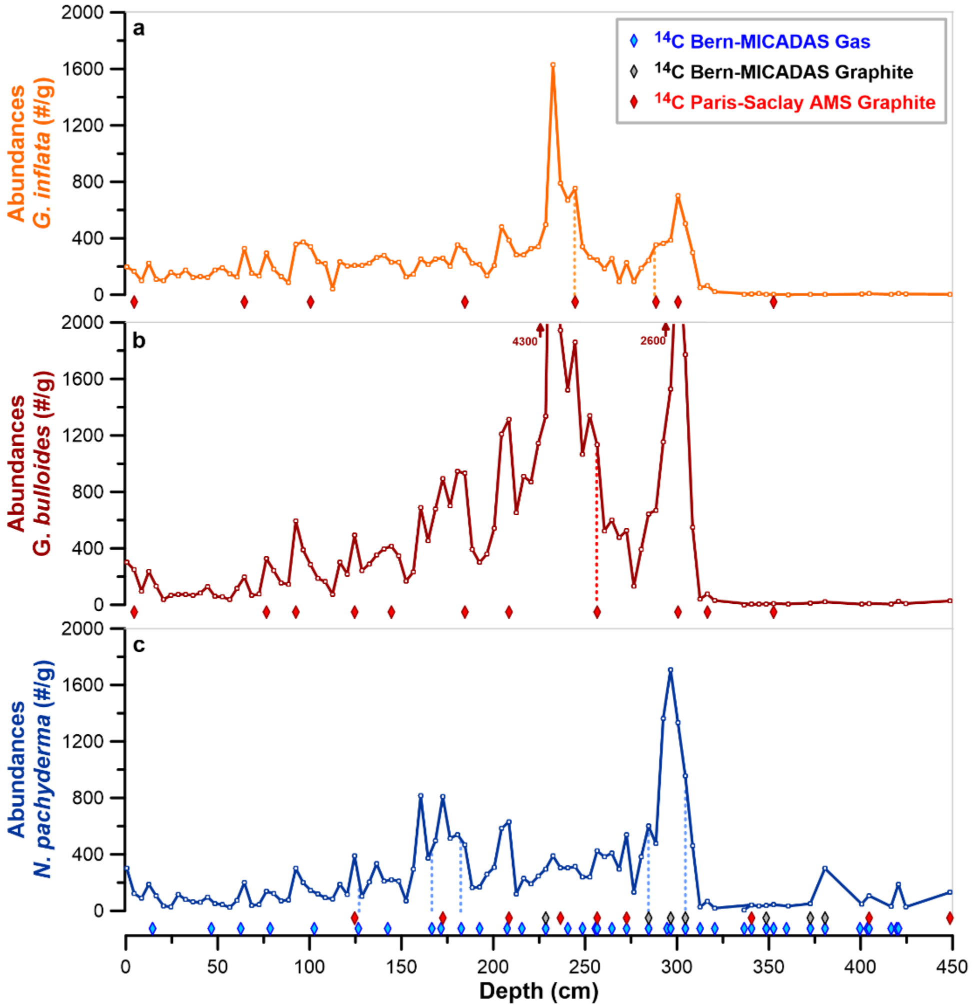

Supplementary Figure 11. Location of planktic foraminifer ^{14}C-dated samples in core MD12-3396CQ relative to planktic foraminiferal abundance peaks. Abundances of (a) *Globorotalia inflata*, (b) *Globigerina bulloides*, and (c) *N. pachyderma*. Symbols show locations of foraminiferal samples dated with the Mini Carbon Dating System (MICADAS) at the University of Bern in gaseous (sample sizes <1 mg $CaCO_3$; blue) or solid form (i.e., graphite, sample sizes >1 mg $CaCO_3$; grey) and with the University of Paris-Saclay accelerator mass spectrometer (AMS) as graphite (samples sizes mostly >1 mg $CaCO_3$; red). Depth intervals of samples not selected from abundance peaks (out of necessity) are highlighted by a stippled line.

Supplementary References

1. Park, Y.-H., Roquet, F., Durand, I. & Fuda, J. L. Large-scale circulation over and around the Northern Kerguelen Plateau. *Deep. Res. Part II Top. Stud. Oceanogr.* **55**, 566–581 (2008).
2. Dutkiewicz, A., Müller, R. D., Hogg, A. M. & Spence, P. Vigorous deep-sea currents cause global anomaly in sediment accumulation in the Southern Ocean. *Geology* **44**, 663–666 (2016).
3. Bareille, G., Grousset, F. E., Labracherie, M. & Labeyrie, L. D. Origin of detrital fluxes in the southeast Indian Ocean during the last climatic cycles. *Paleoceanography* **9**, 799–819 (1994).
4. Dezileau, L., Bareille, G., Reyss, J. L. & Lemoine, F. Evidence for strong sediment redistribution by bottom currents along the southeast Indian ridge. *Deep. Res. Part I Oceanogr. Res. Pap.* **47**, 1899–1936 (2000).
5. Key, R. M. *et al.* A global ocean carbon climatology: Results from Global Data Analysis Project (GLODAP). *Global Biogeochem. Cycles* **18**, GB4031–GB4031 (2004).
6. Garcia, H. E. *et al.* World Ocean Atlas 2013, Volume 3: Dissolved Oxygen, Apparent Oxygen Utilization, and Oxygen Saturation. in *NOAA Atlas NESDIS 75* (eds. Levitus, S. & Mishonov, A. V) 27 (2014).
7. Sloyan, B. M. & Rintoul, S. R. The Southern Ocean Limb of the Global Deep Overturning Circulation. *J. Phys. Oceanogr.* **31**, 143–173 (2001).
8. Mantyla, A. W. & Reid, J. L. On the origins of deep and bottom waters of the Indian Ocean. *J. Geophys. Res.* **100**, 2417–2439 (1995).
9. Orsi, A. H., Johnson, G. C. & Bullister, J. L. Circulation, mixing, and production of Antarctic Bottom Water. *Prog. Oceanogr.* **43**, 55–109 (1999).
10. McCartney, M. S. & Donohue, K. A. A deep cyclonic gyre in the Australian-Antarctic Basin. *Prog. Oceanogr.* **75**, 675–750 (2007).
11. Rintoul, S. R. On the origin and influence of Adélie Land Bottom Water. in *Ocean, ice, and atmosphere: Interaction at the Antarctic continental margin, Antarctic Research Series* (eds. Jacobs, S. S. & Weiss, R. F.) **75**, 151–171 (Wiley Online Library, 1998).
12. Whitworth III, T. Two modes of bottom water in the Australian-Antarctic Basin. *Geophys. Res. Lett.* **29**, 1–3 (2002).
13. Kim, J. H. *et al.* Holocene subsurface temperature variability in the eastern Antarctic continental margin. *Geophys. Res. Lett.* **39**, 3–8 (2012).
14. Haddam, N. A. *et al.* Improving past sea surface temperature reconstructions from the Southern Hemisphere oceans using planktonic foraminiferal census data. *Paleoceanography* **31**, 822–837 (2016).
15. Nürnberg, D. Magnesium in tests of *Neogloboquadrina pachyderma* sinistral from high northern and southern latitudes. *J. Foraminifer. Res.* **25**, 350–368 (1995).
16. Locarnini, R. A. *et al.* World Ocean Atlas 2013 Volume 1: Temperature. in *NOAA Atlas NESDIS 73* (eds. Levitus, S. & Mishonov, A. V.) **1**, 40 (Ocean Climate Laboratory, National Oceanographic Data Center, 2013).
17. Jouzel, J. *et al.* Orbital and millennial Antarctic climate variability over the past 800,000 years. *Science* **317**, 793–796 (2007).
18. Paterne, M., Michel, E. & Héros, V. Variability of marine ^{14}C reservoir ages in the Southern Ocean highlighting circulation changes between 1910 and 1950. *Earth Planet. Sci. Lett.* **511**, 99–104 (2019).
19. Olsen, A. *et al.* The Global Ocean Data Analysis Project version 2 (GLODAPv2) – an internally consistent data product for the world ocean. *Earth Syst. Sci. Data* **8**, 297–323 (2016).
20. Olsen, A. *et al.* GLODAPv2.2019 - an update of GLODAPv2. *Earth Syst. Sci. Data* **11**, 1437–1461 (2019).
21. Hogg, A. G. *et al.* SHCAL13 Southern Hemisphere Calibration, 0-50,000 years cal BP. *Radiocarbon* **55**, 1–15 (2013).
22. Soulet, G. Methods and codes for reservoir-atmosphere ^{14}C age calculations. *Quat. Geochronol.* **29**, 97–103 (2015).
23. Bronk Ramsey, C. Deposition models for chronological records. *Quat. Sci. Rev.* **27**, 42–60 (2008).
24. Bereiter, B. *et al.* Revision of the EPICA Dome C CO_2 record from 800 to 600 kyr before present. *Geophys. Res. Lett.* **42**, 542–549 (2015).
25. Marcott, S. A. *et al.* Centennial-scale changes in the global carbon cycle during the last deglaciation. *Nature* **514**, 616–619 (2014).
26. Monnin, E. *et al.* Atmospheric CO_2 concentrations over the last glacial termination. *Science* **291**,

112–114 (2001).
27. Skinner, L. C., Fallon, S., Waelbroeck, C., Michel, E. & Barker, S. Ventilation of the deep Southern Ocean and deglacial CO_2 rise. *Science* **328**, 1147–1151 (2010).
28. Waelbroeck, C. *et al.* The timing of deglacial circulation changes in the Atlantic. *Paleoceanography* **26**, PA3213–PA3213 (2011).
29. Gottschalk, J. *et al.* Biological and physical controls in the Southern Ocean on past millennial-scale atmospheric CO_2 changes. *Nat. Commun.* **7**, 1–11 (2016).
30. Hoogakker, B. A. A., Elderfield, H., Schmiedl, G., McCave, I. N. & Rickaby, R. E. M. Glacial-interglacial changes in bottom-water oxygen content on the Portuguese margin. *Nat. Geosci.* **8**, 40–43 (2015).
31. Ronge, T. A. *et al.* Radiocarbon Evidence for the Contribution of the Southern Indian Ocean to the Evolution of Atmospheric CO_2 Over the Last 32,000 Years. *Paleoceanogr. Paleoclimatology* **35**, 1–16 (2020).
32. Butzin, M., Köhler, P. & Lohmann, G. Marine radiocarbon reservoir age simulations for the past 50,000 years. *Geophys. Res. Lett.* **44**, 8473–8480 (2017).
33. Veres, D. *et al.* The Antarctic ice core chronology (AICC2012): an optimized multi-parameter and multi-site dating approach for the last 120 thousand years. *Clim. Past* **9**, 1733–1748 (2013).
